# Teachers’ post-pandemic outlook on the role of Technological and Pedagogical Content Knowledge in coping with burnout under adverse conditions: How a job demand transformed into a job resource

**DOI:** 10.3389/fpsyg.2023.1129910

**Published:** 2023-03-08

**Authors:** Negar Rastegar, Mehrak Rahimi

**Affiliations:** English Department, Faculty of Humanities, Shahid Rajaee Teacher Training University, Tehran, Iran

**Keywords:** burnout, coping strategies, COVID-19, post-pandemic, TPACK

## Abstract

**Introduction:**

The sudden change of instructional mode from face-to-face to online teaching during the COVID-19 pandemic forced teachers to develop their ICT skills and knowledge to cope with newly imposed job pressures. The imbalance between job demands and resources in this context led to teachers’ severe burnout. This retrospective study utilized a mixed methods approach to examine teachers’ coping mechanisms, Technological and Pedagogical Content Knowledge (TPACK), and job burnout amid the COVID-19 pandemic.

**Methods:**

Data were gathered from 307 teachers on their experience of emergency remote teaching (ERT) at the time they returned to school in the Spring of 2022. Structural Equation Modeling was used to investigate the mediating role of TPACK in the relationship between coping strategies and burnout.

**Results:**

The results revealed direct effects in the pathways of avoidant, active positive, and evasive coping to burnout highlighting the role of avoidant strategies in harming teachers’ well-being and problem-focused strategies in promoting teachers’ mental health. Also, indirect effects of active positive coping to burnout through TPACK, as a constructive approach to fighting back the crisis, were verified. Further, the direct effect of TPACK on burnout as a hindrance was significant, showing that higher levels of TPACK guaranteed lower job burnout and emotional drain. Analyzing interviews with 31 teachers revealed that TPACK functioned as a stressor at the outset of the pandemic and a resource for overcoming the strain and resolving the challenges in the midst of the crisis till schools reopened.

**Discussion:**

The findings underscore the significant role of teachers’ updated knowledge base in reducing their job pressure and taking proper decisions to cope well with unforeseen circumstances. The study has practical implications for policymakers, teacher educators, and school administrators to pay immediate attention to collective wisdom, organizational support, and technological infrastructures for improving teachers’ well-being and professional success.

## 1. Introduction

The prolonged crisis of the COVID-19 pandemic imposed adverse living and working conditions on people from all walks of life and particularly placed intolerable burdens on teaching professionals who have already been evidenced to suffer from severe job burnout in normal conditions. The imbalance between the excessive job demands and insufficient resources that took some time to be redressed caused considerable stress, fatigue, and strain and eventually high levels of job burnout among teachers and educators (Padmanabhanunni et al., 2022).

Job-related causes of burnout during the pandemic have been extensively researched during the last 2 years. While the origins of job burnout like workload and role conflict and ambiguity have already been documented prior to the pandemic (Bakker et al., 2007), their influence on teachers’ job burnout in this period seems to be more profound. The possible justification for this could be the crucial role of technology in sustaining education during lockdowns and the way technology was inextricably interwoven with all educational activities of the teachers, students, and school staff. In normal situations, change on a massive scale like this takes place as “a process through which people and organizations move as they gradually come to understand and become skilled and competent in the use of new ways” (Hall and Hord, 2006, p. 4). *Time* as a key element in the diffusion of new ideas underscores the importance of gradual adoption of change as a process through which an innovation is diffused and normalized within a social system through communication channels (Rogers and Scott, 1997). As a result of this deficient and sudden change, key members of the education sector were pressed into managing a variety of educational technology challenges (Oyedotun, 2020).

In this stressful situation, the teachers continuously appraise and reappraise their troubled relationship with the environment (Lazarus and Folkman, 1984) trying to find the answer to “What can I do” (Lazarus and Folkman, 1984, p. 142) to alleviate the concomitant sufferings of the pandemic. Personal resources such as resilience (e.g., Padmanabhanunni et al., 2022), attitudes (e.g., Daumiller et al., 2021), efficacy (Sokal et al., 2020), and adoption of coping strategies (e.g., Wang et al., 2022) are among the documented efforts to deal with the stressors.

Considering the central role of ICTs in the unprecedented change of face-to-face instruction to online teaching, some technology-related variables including attitudes toward change (Daumiller et al., 2021), previous ICT training (Stang-Rabrig et al., 2022), and Internet/device access and connection quality (Kamal and Illiyan, 2021) have been sparingly examined to shed more light on their contribution to the status of teachers’ mental health during the pandemic. In this framework, very few researchers have shown interest in the interdependence of teachers’ ICT skills and knowledge, job burnout, and coping mechanisms (e.g., Stan, 2022), in spite of the fact that technological and pedagogical content knowledge (TPACK) has been documented to be related to teachers’ technostress in pre-pandemic studies (Kay, 2008; Joo et al., 2016). To address this lacuna in research, the current study assumes a key role for TPACK as one essential resource that can assist teachers in adopting coping strategies to manage their job burnout during the pandemic.

## 2. Review of literature

### 2.1. Teachers’ burnout during the COVID-19 pandemic

Teachers feel emotionally exhausted and extremely drained as a result of the unrelenting pressures of their working condition that has been exacerbated during the pandemic. The sudden shift from face-to-face instruction to online teaching, or emergency remote teaching (ERT) as it is called, generated unprecedented job demands for which the teachers were unprepared and untrained (Etchells et al., 2021). Looking carefully into the lived experiences of teachers during this time is indicative of teachers’ deterioration of mental and physical health (Wakui et al., 2022) exhibiting the symptoms of moderate to high job burnout (Sokal et al., 2020).

Burnout is “a psychological syndrome emerging as a prolonged response to chronic interpersonal stressors on the job” (Maslach and Leiter, 2016, p. 103). Burnout consists of three dimensions of emotional exhaustion, i.e., the feeling of being drained of emotional and physical resources; depersonalization of others, i.e., emotional or cognitive disconnection from work; and loss of personal accomplishment, i.e., the feeling of lacking efficacy, attainment, and productivity in work (Maslach, 2015).

Teachers’ burnout during the pandemic has been examined in relation to two sets of working conditions, that is demands and resources, based on the assumption that “burnout results from high job demands and poor job resources” (Schaufeli and Taris, 2014, p. 46). Adopting new approaches to planning lessons and instructional practices (Honigsfeld and Nordmeyer, 2020), learning to work with new platforms and introducing them to students (Etchells et al., 2021), longer working hours (Kaufman and Diliberti, 2021), and keeping students engaged in online classes (Kaup et al., 2020) are among the job demands teachers faced unexpectedly while organizational resources such as support from the school staff or colleagues were not widely available.

What is most noteworthy here is that both Job-Demands-Resources (JD-R) model (Demerouti et al., 2001) and the three-dimensional view of burnout (Maslach, 2015) offer liberal interpretations of how job burnout and coping strategies are associated. Leiter (1989), presumably backed by Maslach (2015), put forward the proposal that the three dimensions of burnout can be viewed in terms of the stress–strain-coping framework of Lazarus and Folkman (1984). In this way, emotional exhaustion is associated with strain (Leiter, 1989; Maslach, 2015); depersonalization is linked to the notion of coping (Lee and Ashforth, 1990) and personal distancing; and personal accomplishment represents self-evaluation and inefficacy (Maslach, 2015) that is “an outcome of the stress–strain-coping sequence” (Leiter, 1989, as cited in Lee and Ashforth, 1990, p. 744).

In the same vein, in trying to propose a multilevel model of burnout, Bakker and de Vries (2021) elaborate more on how avoidant coping strategies within the framework of self-regulation are maladaptive, and as individuals’ levels of job strain and burnout raise, they become less capable of selecting a coping strategy that suitably matches the demands of the situation. They further underscore the role of coping flexibility as the ability to use diverse coping strategies so that the adjustment to situational demands is fostered to cope with job stress.

### 2.2. Teachers’ coping with job burnout during the COVID-19 pandemic

Coping strategies are “constantly changing cognitive and behavioral efforts to manage specific external and/or internal demands that are appraised as taxing or exceeding the resources of the person” (Lazarus and Folkman, 1984, p. 141). Coping strategies compose different distinctions and groupings as a result of alternative interpretations of coping theoretical underpinnings, psychometric research, or different labeling of the same strategies. Within the appraisal model, coping strategies are of two types: problem-focused coping strategies used to alter the pressure by doing something about it; and emotion-focused used to regulate the negative feelings toward the source of stress. With the rationale that coping strategies are of a more complex nature than identified in the appraisal model, the multidimensional model of coping strategies has identified distinct aspects of problem-focused and emotion-focused coping strategies (Carver et al., 1989). Coping strategies can be grouped into engagement (approach) and disengagement (avoidant) strategies as well (Carver and Connor-Smith, 2010). Approach coping strategies assist in dealing with the stressor and related emotions (e.g., acceptance, positive reframing, active coping), whereas avoidant strategies are used to escape from the threat or related emotions (e.g., denial, self-distraction, and venting).

The association between coping strategies and job burnout, stress, or well-being among healthcare workers has been extensively researched during the pandemic, but empirical work in this regard is scant in the domain of education. The findings of a few studies done on the subject support positive correlations of psychological outcomes (well-being, health, happiness, resilience, and growth during trauma) with approach coping and their negative correlations with avoidant coping among an international sample of teachers (MacIntyre et al., 2020). Nigerian teachers’ burnout is reported to be positively related to their emotion-focused, problem-focused, and dysfunctional coping strategies (Ozoemena et al., 2021), while Iranian teachers’ apprehension has been found to be negatively influenced by approach coping strategies and positively by avoidant coping strategies (Nazari et al., 2022). In their study of Canadian practicing teachers, Wang et al. (2022) reported that teachers who are classified as adaptive copers have shown the highest levels of enjoyment in teaching and job satisfaction, the lowest levels of negative emotions in teaching (anxiety, anger), and the lowest levels of burnout (emotional exhaustion) and quitting intentions. Examining the coping strategies and mental health across three groups of distressed, moderately stressed, and self-efficacious South African teachers, Marais-Opperman et al. (2021) reported a significant difference among them: teachers with a distressed profile who used self-blame as a coping strategy had poor mental health, while those with a self-efficacious profile and religion as a coping strategy had better mental health.

What seems to be neglected in this research arena is the role of teachers’ technological skills and knowledge in their job burnout during the pandemic and its relation to adopting coping strategies. It is assumed that the knowledge of ICTs assisted teachers in coping with their stress and depression and enabled them to maintain their well-being and sustain their students’ education in the midst of the crisis.

### 2.3. Emergency remote teaching and teachers’ ICT knowledge and skills

Unarguably, *technology* is the crux of the issues educationalists, students, and parents dealt with during the COVID-19 pandemic. Central to this recurrent theme of research is teachers’ way of handling online classes by demonstrating their technological knowledge and skills. In the condition that all educational services were given and received through the screen of computers or mobile phones, the quality of education was largely judged through the lens of teachers’ mastery of holding and managing online classes.

Teachers’ body of knowledge, competencies, and skills of integrating technology within teaching is framed in TPACK scheme (Koehler and Mishra, 2009). Being built on Shulman’s (1986) construct of pedagogical content knowledge (PCK), TPACK is a complex interaction of three layers of knowledge, i.e., technology, pedagogy, and content (Yurdakul et al., 2012); and the creation of the knowledge that goes beyond these three separate knowledge bases (Koehler et al., 2013). There are seven components in the TPACK framework including technological knowledge (TK), pedagogical knowledge (PK), content knowledge (CK), pedagogical content knowledge (PCK), technological pedagogical knowledge (TPK), technological content knowledge (TCK), and TPACK. TPACK is a practical framework for both describing the type of knowledge the teachers are required to have to integrate technology into teaching a given content using appropriate instructional approaches and technologies, and delineating how they can develop this knowledge base (Schmidt et al., 2009).

Technological and Pedagogical Content Knowledge is one of the widely researched areas of educational technology. The main themes of related research include model evolution, TPACK for specific subject domains, teachers’ beliefs about TPACK, TPACK measures (Voogt et al., 2013), and the association between TPACK and teachers’ personal characteristics (Cheng and Xie, 2018). In this framework, the reciprocal relationship between TPACK and technostress seems to have a wider sphere of influence on technology use in teachers’ instructional practices as technostress has been reported to be inversely linked to teachers’ TPACK (Ozgür, 2020) and notably, TPACK has significant effects on teachers’ technostress (Joo et al., 2016).

Technological and Pedagogical Content Knowledge as an internal resource (Joo et al., 2016) and one of the key factors to cope with technology-induced psychological stress (Ozgür, 2020) is expected to reduce job burnout, a part of which is constructed by technostress. Technostress is a strong sense of anxiety created as a result of the “inability to cope with the new computer technologies in a healthy manner” (Brod, 1984, p. 16). This negative feeling about technology use is reported to be inversely associated with teachers’ job satisfaction and efficacy (Lee and Lim, 2020), ICT integration (Rahimi and Yadollahi, 2011), and intention to use technology (Joo et al., 2016).

A few studies that have examined the role of teachers’ technological self-efficacy and skills in their job well-being or burnout have yielded mixed findings. Stang-Rabrig et al. (2022), for instance, investigated the relations of teachers’ occupational well-being (stress, exhaustion, job satisfaction) with job resources (e.g., support from colleagues), job demands (e.g., technical difficulties), and personal resources (e.g., self-efficacy with digital media). Surprisingly, the findings revealed positive relations between teachers’ previous ICT usage and perceived stress and exhaustion. In another study, Stan (2022) examined the additional value of TPACK in explaining the relation between job burnout and job-related affective well-being over and above personality traits. The result showed that teachers’ TPACK was negatively related to job burnout and positively related to well-being.

This puzzling paradox puts teachers’ TPACK under the spotlight as having either a facilitative or a debilitative role in the relationship between teachers’ coping mechanisms and job burnout during the pandemic. Day et al. (2019) have earlier noted the potential paradox of ICTs and worker well-being in terms of ICT autonomy, social connectivity, and productivity within iParadox Triad scheme. This means that ICTs can be both a job demand and resource depending on teachers’ appraisal of the strain, the construal of job burnout in the context of stress, and the deployed coping strategies. Given the scarcity of research in this domain, the current study examines the role of TPACK in the relationship between teachers’ coping mechanisms and their job burnout during the pandemic. The originality of this study is reflected in its design and the way the variables are studied and measured. The retrospective nature of the study demands the participants’ contemplation of how they went through the crisis and sustained their professional success amid adverse conditions. The mixed methods approach urges both quantitative and qualitative data gathering and analysis and thus leads to a more in-depth understanding of the interrelationship among the variables.

## 3. The structural model

**Figure 1:**
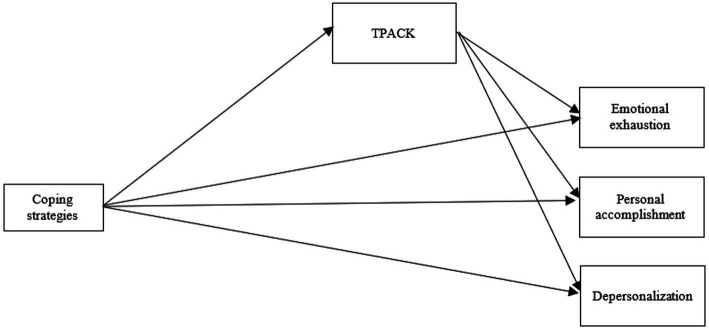
The model of the study.

In the current study, the role of TPACK in teachers’ coping strategies with job burnout amid the COVID-19 pandemic is given careful scrutiny by conducting Structural Equation Modeling (SEM). The structured model and the direct and indirect pathways are depicted in Figure 1.

Quantitative data would be gathered and analyzed to verify the proposed model and answer the following research questions:

1. Is there any significant relationship between teachers’ coping strategies, TPACK, and job burnout?
2. Does teachers’ TPACK function as a mediator in the relationship between their coping strategies and job burnout?

To triangulate the quantitative data, teachers’ post-pandemic outlook would be recorded and analyzed to qualitatively answer a third research question:

3. What are teachers’ perceptions of the role of TPACK in coping with job burnout at the time of the COVID-19 pandemic?

## 4. Method

### 4.1. Participants

Three hundred and fourteen Iranian teachers who were teaching full-time during the COVID-19 crisis participated in the current study. The sample was recruited based on convenience sampling and teachers participated in the study voluntarily. All participants signed the consent form before completing the scales. They were informed of the content of the questionnaires and the goal of the research before going through the scales. The data were gathered in the Spring 2022 when the schools reopened and the teachers had already begun face-to-face teaching.

Upon checking the data, seven participants were removed due to their incomplete answers. The sample included both male (*n* = 119, 38.8%) and female (*n* = 188, 61.2%) teachers who ranged in age from 22 to 66 and had an average experience of 14 years.

### 4.2. Instruments

The following instruments were used to gather the quantitative data for the current study:

- Maslach Burnout Inventory-Educator’s Survey
- TPACK-deep scale
- Brief-COPE scale

Maslach Burnout Inventory-Educator’s Survey (MBI-ES): Teachers’ burnout during the COVID-19 crises was assessed by MBI-ES (Maslach et al., 1996). MBI-ES has 22 items and evaluates teachers’ job burnout with respect to three aspects of burnout syndrome including emotional exhaustion (9 items), personal accomplishment (8 items), and depersonalization (5 items). The items are anchored on a 7-point Likert scale from 0 (never) to 6 (every day).

The reliability of the scale has been reported by several studies since its development across different research contexts with participants of diverse ethnicity. MBI-ES has been validated with Persian samples and its psychometric characteristics were reported to be satisfactory (Pourshahbaz, 2016).

Minor modifications were made to the wording of the scale to make it suitable for the goal of the study that is probing into teachers’ job burnout during ERT when schools were closed as a result of the COVID-19 national lockdown. The factor structure of the scale was put to test and the results confirmed the presence of three components with eigenvalues exceeding 1.0 explaining a total of 54.763% of the variance. The item loadings on each component were exactly similar to the original MBI-ES (Appendix 1). The reliability indices of the three dimensions of MBI-ES for this study are reported in Table 1.

**Table 1:** Reliability indices of the scales.

Variable	Number of items	Cronbach’s alpha (*α*)
*Job burnout*	22	–
Emotional exhaustion	9	0.91
Personal accomplishment	8	0.85
Depersonalization	5	0.75
*TPACK*	33	0.96
Design	10	0.91
Exertion	9	0.90
Knowledge empowerment	3	0.87
Ethics	6	0.85
Proficiency	5	0.84
*Brief-COPE*	28	–
Avoidant	6	0.78
Active positive	6	0.76
Support	4	0.74
Acceptance	2	0.70
Religion	2	0.71
Humor	2	0.70
Self-blame	2	0.73
Evasive	4	0.66

Technological and Pedagogical Content Knowledge-deep: In order to assess the participants’ TPACK, the TPACK-deep scale (Yurdakul et al., 2012) was used. The original TPACK-deep has four subscales including design (10 items), exertion (12 items), ethics (6 items), and proficiency (5 items). The questionnaire anchors on a 5-point Likert scale from 1 (strongly disagree) to 5 (strongly agree).

Technological and Pedagogical Content Knowledge-deep was examined for its factor structure and the result approved a 5-factor model that explained 61.7% of the variance of the construct. All items loaded exactly on the same factors reported by the developers of the TPACK-deep, with one difference. Three items of factor exertion loaded on a fifth factor and due to their common theme and similarities, the factor was labeled “knowledge empowerment” (Appendix 2). The reliability indices of TPACK-deep and its five components for this study are reported in Table 1.

Brief-COPE: Brief-COPE is the brief form of COPE inventory (Carver et al., 1989). This version of the scale was developed because the original scale was long and redundant (Carver, 1997). The Brief-COPE includes 28 items with 14 factors that measure “conceptually differentiable coping reactions” (Carver, 1997, p. 98). The scale can be used in many different ways for retrospective/concurrent situational goals by changing the phrasing of response options (Carver, 1997). The participants were asked to complete the scale expressing the ways they had managed their job burnout during the COVID-19 pandemic.

The result of factor analysis of the scale yielded eight factors that explained a total of 61.54% of the variance (Appendix 3). Similar to the original scale, acceptance, religion, humor, and self-blame remained as two-item factors with the same items of the original scale loading on each factor. Four higher-order factors emerged that had remarkable consistency with the original scales (Carver et al., 1989; Carver, 1997) including avoidant coping (subscales: denial, substance use, and behavioral disengagement), active positive coping (subscales: active coping, positive reframing, planning), support (subscales: emotional support, instrumental support), and evasive coping (self-distraction, venting). The reliability indices of the factors of Brief-COPE are reported in Table 1.

The interviews: With the intention of triangulating, 31 teachers were asked to take part in a structured interview. The questions of the interview targeted the personal stress and professional challenges the teachers faced during online teaching amid the COVID-19 pandemic; how they managed those problems; and if they received any support from colleagues or administrators to do so.

### 4.3. Procedure

Upon reviewing the literature and understanding the gaps, the hypothetical model of the study was devised and suitable instruments were selected. All instruments were piloted and their validity and factor structure were examined (See Appendices 1–3). The scales were then distributed among 314 teachers who experienced working under adverse conditions during the COVID-19 pandemic when the schools were closed from February 2020 to March 2022. Before completing the scales, a written and brief description of the research project and the scales were given to the participants. A consent form was also provided and upon signing the agreement, the teachers were asked to fill in the scales.

The quantitative data were checked and incomplete questionnaires were removed. The data then were inserted into the data analysis software programs. Suitable quantitative data analysis techniques were applied and the obtained results were interpreted.

To have a deeper insight into the relationship between the variables of the study, the teachers were asked for voluntary participation in the interviews. Thirty-one teachers agreed to take part in the interviews and their answers to five questions were analyzed by content analysis, both manually and using computer software.

### 4.4. Data analysis

Adopting a mixed methods approach, two sets of data analyses were used to attain the goals of the study and shed light on the interplay between TPACK, coping strategies, and job burnout.

Quantitative data analysis including descriptive and inferential statistics was used to answer research questions number 1 and 2. Descriptive statistics provided an understanding of the data in terms of characteristics and distribution of the values. SEM was used to scrutinize the mediating role of TPACK in the relationship between coping strategies and job burnout. SEM “is a statistical method that examines the relationships among numerous variables in a simultaneous way” (Collier, 2020, p. 1) and thus a suitable data analysis to test the hypothesized model of this study (Figure 1). For quantitative data analysis, IBM SPSS Statistics and Analysis of Moment Structures (AMOS) software were used.

Qualitative data analysis included content analysis of teachers’ answers to the interview questions. Both manual and computer-assisted coding were used to analyze the data. Computer-assisted coding was done with NVivo12 Pro.

## 5. Results

### 5.1. Quantitative data analysis

Descriptive statistics of the variables: Descriptive statistics of the variables of the study are presented in Table 2. Examining the descriptive statistics of the coping strategies shows that teachers’ most frequently used coping strategies to manage burnout during COVID-19 were acceptance (Mean = 2.094, SD = 0.687) and active positive coping (Mean = 2.089, SD = 0.551) on a scale of 0–3. The teachers reported that they were not fond of avoidant strategies (Mean = 0.849, SD = 0.646).

**Table 2:** Descriptive statistics of the variables of the study.

Variable	Mean	SD
*Coping strategies*	–	–
Avoidant coping	0.849	0.646
Active positive coping	2.089	0.551
Support	1.734	0.680
Acceptance	2.094	0.687
Religion	1.812	0.861
Humor	1.345	0.844
Self-blame	1.377	0.850
Evasive	1.614	0.586
*TPACK*	3.780	0.679
Design	3.830	0.750
Exertion	3.747	0.788
Knowledge empowerment	4.293	0.787
Ethics	3.675	0.797
Proficiency	3.556	0.830
*Job burnout*	–	–
Emotional exhaustion	27.873	10.175
Personal accomplishment	18.384	7.709
Depersonalization	13.589	4.971

On average, the participants reported being well capable of using technology to empower themselves during the COVID-19 pandemic (Mean = 4.293, SD = 0.787) and planning online classes relying on their technological and pedagogical knowledge (Mean = 3.83, SD = 0.75) on a scale of 1–5.

Based on the MBI-ES scoring guideline (Schaufeli et al., 1996), the participants displayed a high level of burnout on emotional exhaustion (Mean = 27.873, SD = 10.175) and depersonalization (Mean = 13.589, SD = 4.971) but a low level of burnout on personal accomplishment (Mean = 18.384, SD = 7.709) during the COVID-19 pandemic and ERT.

Inter-correlations between the variables: Table 3 shows the correlations between the variables of the study including coping strategies, TPACK, and burnout dimensions.

**Table 3:** Inter-correlations between the variables of the study.

	**1**	**2**	**3**	**4**	**5**	**6**	**7**	**8**	**9**	**10**	**11**	**12**
1. Avoidant	1											
2. Active	−0.013	1										
3. Support	0.209^**^	0.499^**^	1									
4. Acceptance	−0.066	0.502^**^	0.302^**^	1								
5. Religion	0.145^*^	0.473^**^	0.428^**^	0.373^**^	1							
6. Humor	0.345^**^	0.240^**^	0.273^**^	0.207^**^	0.255^**^	1						
7. Self-blame	0.404^**^	0.065	0.276^**^	−0.038	0.146^*^	0.217^**^	1					
8. Evasive	0.350^**^	0.439^**^	0.488^**^	0.239^**^	0.372^**^	0.306^**^	0.302^**^	1				
9. TPACK	−0.098	0.330^**^	0.140^*^	0.255^**^	0.140^*^	0.133^*^	−0.073	0.102	1			
10. EE	0.274^**^	−0.103	0.130^*^	0.001	0.067	0.080	0.153^**^	0.211^**^	−0.242^**^	1		
11. PA	0.129^*^	−0.249^**^	−0.082	−0.179^**^	−0.097	−0.099	0.144^*^	0.031	−0.536^**^	0.296^**^	1	
12. DP	0.593^**^	−0.054	0.130^*^	−0.062	0.109	0.270^**^	0.286^**^	0.322^**^	−0.196^**^	0.472^**^	0.243^**^	1

Considering the association between coping strategies and burnout dimensions, it is found that avoidant and self-blame strategies are positively related to all dimensions of burnout; while support and evasive coping strategies are positively related to two dimensions of burnout, that is, emotional exhaustion and depersonalization. Active coping and acceptance are negatively related to personal accomplishment; and humor is positively related to depersonalization.

As for the relationship between coping strategies and TPACK, it is revealed that active positive, support, acceptance, religion, and humor are positively related to TPACK. TPACK was found to be negatively related to all dimensions of burnout.

The measured model: AMOS software and the maximum likelihood estimation (MLE) procedure were used to do the required data analyses and test the model. Table 4 illustrates the fit indices of the hypothesized model including chi-square (*χ*^2^), root-mean-square error of approximation (RMSEA), root-mean-square residuals (RMR), standardized root-mean-square residuals (SRMR) comparative fit index (CFI), the goodness-of-fit index (GFI), and adjusted goodness-of-fit index (AGFI); and their acceptable fit indices (Schermelleh-Engel et al., 2003). As Table 4 shows, all model fit statistics were within the acceptable ranges (*ꭓ*^2^/sd = 2.97, RMSEA = 0.066, RMR = 0.033, SRMR = 0.039, NFI = 0.93, CFI = 0.95, GFI = 0.94, and AGFI = 0.85).

**Table 4:** Evaluation of the fit indices for model testing.

Index	Model	Perfect fit	Good or acceptable fit	Decision
*ꭓ*^2^/sd	2.97	*ꭓ*^2^/sd ≤ 3	*ꭓ*^2^/sd ≤ 3	Good fit
RMSEA	0.066	RMSEA ≤ 0.05	RMSEA ≤ 0.08	Good fit
RMR	0.033	RMR ≤ 0.05	RMR ≤ 0.08	Perfect fit
SRMR	0.039	SRMR ≤ 0.05	SRMR ≤ 0.08	Perfect fit
NFI	0.93	NFI ≥ 0.95	NFI ≥ 0.90	Good fit
CFI	0.95	CFI ≥ 0.95	CFI ≥ 0.90	Perfect fit
GFI	0.94	GFI ≥ 0.95	GFI ≥ 0.90	Good fit
AGFI	0.85	AGFI ≥ 0.90	AGFI ≥ 0.85	Good fit

Table 5 shows the direct effects between the variables of the study. Examining the direct paths from eight coping strategies to TPACK shows that the only significant effect is observed in the path active positive strategies-TPACK (*β* = 0.285, *t* = 3.722, *p* < 0.001).

**Table 5:** Regression coefficients and standard errors for the pathways of the model.

Relationships with	TPACK				Emotional exhaustion				Personal accomplishment				Depersonalization			
Variables	*B*	*β*	S.E	*p*	*B*	*β*	S.E.	*p*	*B*	*β*	S.E	*p*	*B*	*β*	S.E	*p*
Avoidant	−0.087	−0.088	0.066	0.187	0.285	0.163	0.109	**	0.037	0.025	0.085	0.258	0.729	0.474	0.082	***
Active positive	0.330	0.285	0.089	***	−0.444	−0.217	0.149	**	−0.185	−0.106	0.115	0.108	−0.166	−0.092	0.112	0.061
Support	−0.002	−0.002	0.066	0.975	0.176	0.106	0.110	0.071	−0.041	−0.029	0.085	0.814	−0.045	−0.031	0.082	0.609
Acceptance	0.087	0.094	0.061	0.154	0.154	0.094	0.102	0.311	0.006	0.004	0.079	0.514	−0.010	−0.007	0.076	0.590
Religion	−0.011	−0.015	0.049	0.823	0.044	0.033	0.082	0.522	−0.007	−0.006	0.063	0.973	0.107	0.029	0.061	0.579
Humor	0.068	0.091	0.047	0.146	−0.012	−0.009	0.078	0.689	−0.078	−0.068	0.060	0.087	0.018	0.091	0.059	0.060
Self-blame	−0.059	−0.077	0.048	0.217	−0.008	−0.006	0.079	0.842	0.092	0.081	0.061	0.058	0.033	0.015	0.059	0.565
Evasive	−0.015	−0.013	0.077	0.849	0.369	0.191	0.127	**	0.220	0.134	0.099	*	0.317	0.187	0.095	***
TPACK	−	−	−	−	−0.432	−0.244	0.105	***	−0.786	−0.520	0.087	***	−0.255	−0.164	0.078	***

^*^p < 0.05; ^**^p < 0.01; and ^***^ < 0.001.

Examining the direct paths from eight coping strategies to emotional exhaustion shows that three paths are significant including avoidant-emotional exhaustion (*β* = 0.163, *t* = 2.609, *p* < 0.01), active positive-emotional exhaustion (*β* = −0.217, *t* = 2.980, *p* < 0.01), and evasive-emotional exhaustion (*β* = 0.191, *t* = 2.894, *p* < 0.01). Examining the direct paths from eight strategies to personal accomplishment shows that the only significant path is evasive-personal accomplishment (*β* = 0.134, *t* = 2.230, *p* < 0.05). Examining the direct paths from eight strategies to depersonalization shows that two paths are significant including avoidant-depersonalization (*β* = 0.474, *t* = 2.609, *p* < 0.001) and evasive-depersonalization (*β* = 0.187, *t* = 3.318, *p* < 0.001).

Examining the direct paths from TPACK to dimensions of burnout displays that all paths including TPACK-emotional exhaustion (*β* = −0.244, *t* = 4.124, *p* < 0.001), TPACK-personal accomplishment (*β* = −0.52, *t* = 9.083, *p* < 0.001), and TPACK-depersonalization (*β* = −0.164, *t* = 3.275, *p* < 0.001) are significant.

In Table 6, the regression coefficients that resulted from the mediation analysis are presented. The results revealed that active positive coping strategies had a significant indirect influence on all three dimensions of job burnout, that is emotional exhaustion (*β* = −0.142, *p* < 0.001), personal accomplishment (*β* = −0.259, *p* < 0.01), and depersonalization (*β* = −0.084, *p* < 0.001) via TPACK.

**Table 6:** Direct, indirect, and total effects for the mediation using a Bootstrap Analysis with 95% Confidence Interval.

Relationships with	Emotional exhaustion					Personal accomplishment					Depersonalization				
Variables	Direct effect	Indirect effect through TPACK	Confidence interval		Total effect	Direct effect	Indirect effect through TPACK	Confidence interval		Total effect	Direct effect	Indirect effect through TPACK	Confidence interval		Total effect
			Low	High				Low	High				Low	High	
Avoidant	0.285^**^	0.037	−0.014	0.115	0.323^**^	0.037	0.068	−0.039	0.199	0.105	0.729^**^	0.022	−0.006	0.085	0.751^**^
Active positive	−0.444^**^	−0.142^***^	−0.311	−0.052	−0.587^**^	−0.185	−0.259^**^	−0.424	−0.105	−0.444^**^	−0.166	−0.084^**^	−0.206	−0.030	−0.250
Support	0.176	0.001	−0.053	0.069	0.177	−0.041	0.002	−0.100	0.111	−0.040	−0.045	0.001	−0.036	0.043	−0.045
Acceptance	0.154	−0.038	−0.127	0.014	0.117	0.006	−0.069	−0.202	0.030	−0.063	−0.010	−0.022	−0.087	0.007	−0.032
Religion	0.044	0.005	−0.034	0.058	0.049	−0.007	0.009	−0.067	0.096	0.002	0.033	0.003	−0.017	0.045	0.036
Humor	−0.012	−0.030	−0.093	0.005	−0.041	−0.078	−0.054	−0.144	0.020	−0.131	0.107	−0.017	−0.064	0.004	0.090
Self-blame	−0.008	0.025	−0.013	0.079	0.017	0.092	0.046	−0.029	0.123	0.139	0.018	0.015	−0.007	0.051	0.033
Evasive	0.369^**^	0.006	−0.048	0.074	0.375^**^	0.220^*^	0.011	−0.091	0.123	0.231^*^	0.317^**^	0.004	−0.028	0.047	0.320^*^
TPACK	−0.432^**^	---	−	−	−0.432^**^	−0.786^**^	−	−	−	−0.786^**^	−0.255^**^	−	−	−	−0.255^**^

^*^p < 0.05; ^**^p < 0.01; and ^***^p < 0.001.

**Figure 2:**
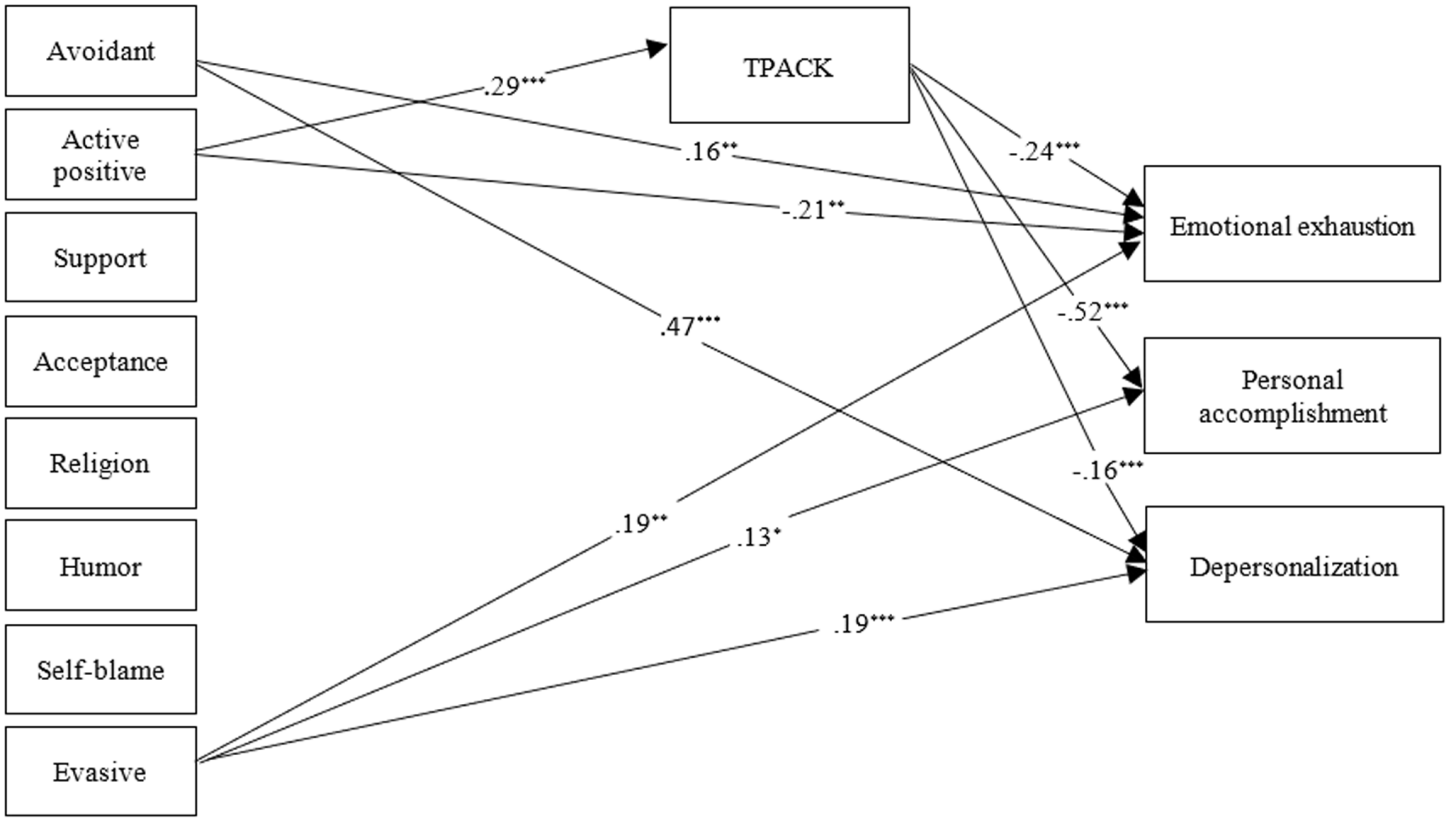
The structural equation modeling (SEM) of the interrelationship between the variables (Regression coefficients are standardized).

The direct and indirect pathways of the tested model are shown in Figure 2. Only the significant paths are depicted.

### 5.2. Qualitative data analysis

The data gained from the interviews were first analyzed manually and then inserted into the software NVivo12 Pro for establishing the themes and sub-themes. The coding was done in three steps of pre-coding, first-cycle coding, and second-cycle coding (Saldana, 2016).

In pre-coding, both researchers read the transcripts meticulously several times contemplating the issues the respondents raised. In the first-cycle coding, the texts were coded by the two researchers. Then the codes were compared and contrasted and in case of any disagreements, the issues were discussed and resolved. The intercoder Kappa was calculated to ascertain reliability. In the second-cycle coding, the themes and subthemes were developed based on the first coding cycle. The data then were inserted into NVivo12 Pro and the hierarchy of codes, themes, and subthemes was established (Table 7).

**Table 7:** Themes and subthemes.

Themes	Rank	Word	Count	Weighted percentage (%)	Subthemes	Meaning
Sources of burnout	1	students	97	5.50	Students, Technology, Workload, Personal issues	Causes of teachers’ burnout during the pandemic
	2	teaching	46	2.61		
	3	Internet	27	1.53		
	4	work	20	1.13		
	5	communication	19	1.08		
	6	content	19	1.08		
Coping mechanisms	1	students	34	3.47	Students, Technology	Teachers’ ways of coping with the main stressors
	2	tried	20	2.04		
	3	teaching	18	1.83		
	4	knowledge	15	1.53		
	5	content	13	1.33		
	6	software	12	1.22		
Support	1	teaching	36	2.41	Administrative, Social, Technological	The available resources
	2	Internet	34	2.27		
	3	provide	32	2.14		
	4	students	28	1.87		
	5	support	27	1.80		
	6	colleagues	26	1.74		

The result of the analysis of the interviews regarding theme 1, i.e., burnout, revealed four subthemes including students, technology, workload, and personal issues. The subtheme “students” had eight categories, the theme “workload” and “technology” had two categories each, and the theme “personal issues” had three categories (Table 8).

**Table 8:** Theme 1 sources of burnout: Subthemes, categories, and example quotations.

Subtheme	Example quote
*Students*
• Motivation	“The students, even though I appeared in the classroom with a high level of energy and I also tried to explain the subject matter perfectly, used to attend the classroom only to announce their presence and without any interest and motivation.”
• Engagement and interaction	“The biggest challenge was trying to engage students in classroom activities when using SHAD or WhatsApp. Almost half of the students would vanish as soon as the class started and it seemed like you were teaching in an empty classroom.”
• Attention and learning	“I saw my students did not learn much from these online classes because they were not present in the class while simultaneously acing their tests; and I was worried about their next year when the pandemic was finally over and they had not learned anything.”
• Class management/discipline	“Students, knowing that the teacher does not have physical supervision during the class, were doing various things during the class.”
• Cheating	“In virtual space, students could easily communicate with each other and this communication did not allow the teacher to assess the real level of students’ knowledge.”
*Technology*
• TPACK	“We were under a lot of pressure, and many times we felt that we were burnt out because we could not find a practical way to solve students’ problems.” “I can boldly claim that my biggest challenge was my lack of distance teaching knowledge because we were not taught how to teach in such conditions.”
• Technological infrastructures	“The weak Internet in the places where my students lived caused the class process to be cut off and they did not attend the class on time or did not send the assignments on time.”
*Workload*
• e-Leaning content	“Creating content for virtual classes could be time-consuming and sometimes you would spend so many hours creating a clip but when you look back at it you see that it does not have any sound because your headphone was broken and you did not know it, it happened to me once and I instantly burst into tears.”
• Time	“It seemed that I could find no more free time for myself. I had to spend all my day doing school stuff like creating video clips or designing an online test.”
*Personal issues*
• Financial issues	“The teachers had to pay the related expenses themselves.”
• Privacy violation	“All parents and students had access to the teacher’s phone number, and many times they disturbed the teacher with occasional calls and made unusual requests.”
• Physical health damage	“My eyesight became very poor and suffered a lot because I had to constantly look at the phone and follow the teaching and learning process of the students.”

Within theme 2, i.e., coping mechanism, two subthemes of students and technology emerged. The subtheme “students” had eight categories and the subtheme “technology” had two categories (Table 9).

**Table 9:** Theme 2 coping mechanisms: Subthemes, categories, and example quotations.

Subtheme	Example quote
*Students*
• Motivation	“Motivating the students by making them participate in activities and also creating competition between them, for example, I asked them to prepare podcasts and videos or audio files and send them to the lesson group.”
• Engagement and interaction	“Asking questions from my students constantly during teaching was the best way to conquer the challenge of not knowing if they are present in my classroom.”
• Attention and learning	“In the second semester of the pandemic, I decided to get students more involved in class time. Near the end of each session, I gave them some assignments to do at home and send their answers to my Telegram or WhatsApp account.
• Class management and discipline	“Creating diverse activities to attract more students’ attention and prevent disorder in the classroom. For example, using different pictures, animations, and clips to teach each part of the lesson, as well as asking various questions related to the previous lesson to start the new lesson and increase the participation of students.”
• Cheating	“I designed different tests with different answers so that not every question would be the same for every student.” “I used online tests to reduce student cheating during the exam.”
*Technology*
•TPACK	“The first months of my job was like a trial-and-error process. I tried to search and update myself by taking part in workshops, watching videos in YouTube.” “I have tried a lot of software for making videos to increase their quality and decrease their size.” “I learned from my colleagues about how to manage online classes.”
• Technological infrastructures	“I bought a modem with higher performance and speed as well as a smartphone with a higher memory at my own expense.” “I also tried to prepare course content in advance and send it to student groups when the Internet speed problems were less.”

With respect to theme 3, i.e., support, three subthemes of administrative, social, and technological support emerged. The subthemes “administrative support” and “technological support” did not include any categories, yet the subtheme “social support” had two categories (Table 10).

**Table 10:** Theme 3 support: Subthemes, categories, and example quotations.

Subtheme	Example quote
Administrative
	“Unfortunately, training classes for teachers were not held at all.” “There was no special support from the school and the MOE. They were just checking if the classes were held.”
Social
Helpful	“They created some kind of WhatsApp group and channels, in which there were lots of instructional materials regarding for example how to reduce the size of your videos and how to upload them into SHAD and some materials like this.” “The interaction between the teachers made us share our experiences in different fields and this was a basic help in some cases.”
Not helpful	“The teachers I worked with did not even know how to manage virtual teaching and use new technologies. They could probably work with SHAD at most. Therefore, they had no special experience to share with others.”
Technological
	“There was no financial or non-financial support, and only two or three limited Internet packages were given to the teachers, which did not meet the needs of the teachers and their workload.” “No tools such as smartphones and tablets or accessories such as microphones or phone holders were provided to the teacher. Many students did not even have television at home or lived in villages where there was no Internet coverage at all, and unfortunately, the government could not support any of these cases.”

## 6. Discussion

### 6.1. Coping strategies and job burnout

The findings of the study primarily revealed that avoidant strategies with the subscales of denial, substance use, and behavioral disengagement were predictors of two dimensions of job burnout, that is, emotional exhaustion and depersonalization. In other words, teachers who pushed the reality of the pandemic away, used substances to feel better about the crisis or get through it, and reduced or gave up the effort to accomplish their educational goals the pandemic was interfering with, experienced higher emotional fatigue and depersonalization. The use of avoidant strategies is a sign of teachers’ incapability to get along with the stressful situation of the pandemic that inevitably led to a higher level of emotional drain and defensive behavior. The overwhelming nature of the strain and the multifaceted challenge the teachers faced affected both their personal lives (Spadafora et al., 2022) and professional career (Kupers et al., 2022) and caused a sense of emotional distress and detachment from work for those who could not cope well with these stressors.

Evasive strategies with the subscales of self-distraction and venting were the significant predictors of all three dimensions of burnout and contributed to increased teachers’ emotional drain, depersonalization of others at work, and low sense of accomplishment during the crisis. This suggests that neither ventilating one’s negative feelings nor focusing away from them were conducive to having healthy work conditions during the pandemic. Emotion-focused coping strategies are used when people think they cannot do anything with the situation and they have to endure the stressor. Maladaptive strategies and teachers’ sense of negative emotions and burnout are related (Ozoemena et al., 2021) because these strategies are associated with poorer mental health (Burker et al., 2005) and depression (Pavlov and Limbers, 2022); and their use over time may hinder adjustment to stress situation (Carver et al., 1989).

Active positive strategies with subscales planning, positive reframing, and active coping were found to prevent emotional exhaustion. These strategies are problem-focused and let people evaluate the stressful situation and think about what they want to do by looking at the problem from a positive perspective, and then implementing their constructive plans to manage the stress. As found in previous studies, the use of active coping strategies leads to more well-being and mental health while low coping mechanisms cause prolonged burnout (Eddy et al., 2019).

### 6.2. Coping strategies, TPACK, and burnout

The predictive power of active positive coping strategies over TPACK shows that during the crisis, teachers have used these strategies to alter the source of the stress and adopt a more constructive approach to solve the existing problems (Carver et al., 1989). When people believe that they can do something productive to change the stressful condition, they choose problem-focused strategies, otherwise, they deploy emotion-based strategies (Lazarus and Folkman, 1984). As a result, these strategies have been found to be among the most popular coping strategies used by teachers during the pandemic (Nazari et al., 2022; Rajesh et al., 2022).

Principally, deploying problem-solving strategies is dependent on the resources that are at the disposal of the person or the limitations they face when they decide to use these resources (Lazarus and Folkman, 1984). At the outset of the pandemic, the teachers realized that neither the technological infrastructures of the country nor the teachers themselves were ready for this huge and sudden change. Yet, their attitudes to this change evolved and gradually altered from negative to positive; and from somewhere during the crisis, they perceived this change as a positive challenge that could be exploited for their professional empowerment and development (Daumiller et al., 2021).

Being able to use technologies to manage instructional practices guaranteed more sense of professional efficacy (Abbitt, 2011) and thus lowered teachers’ burnout associated with personal accomplishment. The higher their TPACK became, the more they used technologies (Davaasuren et al., 2021), and the more they felt capable of managing the demands of their job. This is actually reflected in the role of TPACK as a hindrance to all three dimensions of burnout in the current study. In line with a few earlier works, this suggests that teachers with higher levels of TPACK were more confident in online classes and experienced less stress and emotional drain (Stan, 2022). Conversely, lack of knowledge and incapability of working with technological devices raised teachers’ technostress (Arslan et al., 2022), because negative feelings toward technology are the result of the constant pressure of acquiring new technological knowledge and skills (Tarafdar et al., 2007). This anxiety impacted their job burnout (Dahabiyeh et al., 2022) and job satisfaction (Aktan and Toraman, 2022) and in a repeating cycle, teachers activated their appraisal mechanism and turned to their resources including TPACK to be able to cope with that strain (Lazarus and Folkman, 1984).

### 6.3. The mediating role of TPACK in the relationship between coping and burnout

TPACK strengthens the effect of active coping on reducing job burnout meaning that the teachers who used more active positive coping and felt they could manage online teaching, experienced less job burnout because they had higher TPACK. This supports the argument for the interconnectedness of coping and TPACK and how these two variables can explain job burnout altogether.

The finding can be backed by iParadox Triad assumption as it confirms the view that ICTs can be located on a job demand-resource continuum that have both positive and negative effects on work well-being based on individuals’ appraisal system (Day et al., 2019). “On the one hand, they can serve as a useful tool to help achieve work-related (and non-work-related) goals. On the other hand, they appear to introduce new stressors that can negatively affect well-being” (Day et al., 2019, p. 583).

Within this framework and based on the stress–strain-coping scheme (Lazarus and Folkman, 1984; Lee and Ashforth, 1990), TPACK could be a stressor, a positive challenge, or irrelevant in the primary appraisals. Logically, TPACK as a stress reducer is of more value for those teachers who had been trained technologically. This is expected because TPACK development courses are reported to affect teachers’ self-efficacy and ability to overcome barriers against classroom technology integration (Knapp, 2017) and choose suitable teaching methods and classroom management (Aktaş and Özmen, 2020).

In the secondary appraisals, based on teachers’ available resources and support, the TPACK could be a threat to work well-being (i.e., a stressor). In case the demand taxed the resources of the teachers (i.e., their TPACK), it was a stressor (as was for most teachers) that might have resulted in job burnout that demanded a response. Thus, TPACK could have been a negative job demand for those teachers who did not have much training and experience in teaching online or hybrid classes before the pandemic. These teachers were forced to redouble their sustained cognitive effort for integrating technology into their instruction without being provided with worthwhile organizational resources and support. Paradoxically, this can also be a job demand for those teachers who had past experience with educational technology but whose prior ICT skills were not enough or helpful for handling a variety of technological challenges they faced in the ERT. The response of both groups, that is empowering one’s TPACK by spending time and effort, could lower the negative effects of the lack of TPACK (e.g., technostress) and thus contributed to less job burnout.

### 6.4. Teachers’ experiences of the ERT

Sources of burnout: One main source of teachers’ burnout was students’ low motivation and interest in remote learning. Students’ low motivation in virtual learning is mainly related to technological infrastructures and social support from teachers and peers (Tan, 2021). As mentioned in interviews, technological infrastructures were linked to lack or insufficient technology access and availability of technological devices as well as students’ low computer skills. Unfortunately, around a quarter of Iranian students (4 million students) did not own a smartphone or did not have good-quality Internet access to be a member of student network known as SHAD during the pandemic (Khabaronline, 2020). Further, due to the flaws of the educational platform, students’ interaction with the teacher, peers, and the content did not lead to the construction of a good learning atmosphere to improve students’ participation in classes (Sun et al., 2022). The instruction was mostly teacher-fronted and content-centered planned, directed, and delivered by the teacher without prioritizing attitudinal goals in the designed activities (Echeverría et al., 2022).

Another source of teachers’ burnout was their concerns about students’ learning due to their absences, low class participation, and cheating. This emotional drain can be viewed from two perspectives: students’ learning outcomes and teachers’ inefficacy. Teachers were fearful of their students’ low learning gains as they could not have a fair evaluation of their learning. One main reason for this was climbed chances of academic dishonesty in remote learning such as contract cheating in doing the assignments or getting help from others (the Internet or peers) in exams (Erguvan, 2021). Hence, some teachers had a sense of inefficacy, as they were not sure if their efforts were enough, their teachings were influential, and the materials they prepared were useful. This uncertainty and skepticism about “what they do is right,” significantly influenced the rise of teachers’ inefficacy in comparison to the pre-pandemic condition (Weißenfels et al., 2022).

Technology was the dominant theme in teachers’ discussion of burnout during the pandemic. As a matter of fact, all other themes and subthemes had direct or indirect links to technology and its pivotal role in distressing teachers or helping them out of the stressful condition of the ERT. Without a doubt, the Internet speed and its penetration in less privileged areas and villages were stressors number one. Following the Internet, the teachers have raised the issue of the local platform, SHAD, and its inadequacies. The overburdened teachers who had to manage extra works felt even more exhausted because they had to work with an application that could not fulfill the educational needs of teachers or students. In addition to teachers, SHAD was not perceived to be a useful educational network by either the students (Rastegar and Rahimi, 2021) or their parents (IRNA, 2021) and was the main source of fatigue for all.

Related to technology, TPACK was one of the most complicated issues teachers talked about during their interviews. The topics of interest associated with the TPACK-burnout included TPACK as a stressor, particularly in the early months of the pandemic, a job demand that has not been addressed appropriately before the pandemic and not well supported during the pandemic, and a disincentive to the sense of self-efficacy. In the first half of the pandemic, most teachers were very confused about the ERT, its trajectory, how it would proceed, and when it would come to an end. They felt incapacitated by lacking TK, TPK, and TCK to be able to successfully teach in this new condition. The shortage of ICT skills and knowledge for teaching raised teachers’ negative feelings and anxiety to a certain extent (Ozgür, 2020). Without much organizational support, the teachers had to empower themselves technologically by spending their time, energy, and money and this led to even more emotional exhaustion and fatigue (Bakker et al., 2007).

As they repeatedly mentioned, teachers spent much of their time preparing e-content because the national platform did not support synchronous communication. While this asynchronous teaching seemed to be more flexible and more adjustable for those who did not have technological support (Etchells et al., 2021), it had its own flaws. Considering teachers’ TPACK level and computer skills, and the organizational support they could receive, teachers’ emotional exhaustion raised because they became physically and psychologically fatigued in the process of e-content production and sharing (Minihan et al., 2022). Additionally, the teachers were required to spend a lot of time correcting students’ homework, designing online tests, and finding appropriate teaching materials on the Internet that increased their workload significantly in comparison to the pre-pandemic situation (Kaufman and Diliberti, 2021). Iranian teachers’ escalated exhaustion as a result of workload seems to be experienced by other teachers during the pandemic worldwide. This includes work–family conflicts (Sokal et al., 2020), sleep reduction or disorder (Barbosa et al., 2022), and health problems such as poor eyesight (Dossari et al., 2022) or backache (Barbosa et al., 2022).

Coping mechanisms: As can be supported by the descriptive statistics, the use of two types of coping strategies, that is active positive coping and acceptance, was prevalent among teachers during the pandemic. These strategies were mainly deployed to cope with challenges the teachers had with their students and technological issues.

Teachers’ frequent use of problem-based coping strategies is a sign of their concern about their students’ learning (Pogere et al., 2019) and how determined they were to find a way to manage their issues. The use of acceptance as a functional strategy also helped them to accept the reality of the pandemic and become engaged in the attempt to deal with this stressful situation (Carver et al., 1989). As a result of that, they performed different courses of action from empowering their knowledge base and mastery of working with technology to implementing efficacious teaching methods and designing good-quality contents and tests. This upskilling raised teachers’ confidence and expertise in online teaching and thus generated a more positive classroom atmosphere that affected students’ learning (Almerich et al., 2016) and acceptance of technology-enhanced instruction (Bostan and Şener, 2021).

Teachers’ concerted efforts and perseverance to empower themselves technologically and develop their TPACK show how cleverly these teachers employed their active positive coping from planning, to positive reframing to active coping strategies. Teachers in the very first weeks of the pandemic realized that pre-pandemic ICT skills and knowledge were one of the most reliable sources of coping with the challenges of the pandemic (Stang-Rabrig et al., 2022). Those who lacked that proficiency or felt less updated in their ICT skills, showed more clear thinking and extra energy (Allen et al., 2020) and acquired or developed their TPACK first by trial and error and searching the Internet, then more systematically by taking part in online classes or seeking help from their more proficient colleagues. Teachers’ talking about their technostress, TPACK, efficacy, and burnout shows that the more they had the experience of working with technology confidently, the more efficacious they became, and thus the more they felt satisfied and happy (Lee and Lim, 2020). As a result, in the second half of the pandemic, the role of TPACK changed from a job stressor to a job resource that could buffer the impact of other demands on burnout itself (Bakker et al., 2005).

Teachers’ adoption of approach coping shows that Iranian teachers had enough determination and capacity to perform their job duties despite the disastrous effects of the pandemic on their life and work conditions. Teachers’ perceptions of the way they could adapt to the change successfully are indicative of their internal and interpersonal resilience as they upskilled themselves in both technology and pedagogy to guarantee efficient teaching and learning processes (Raghunathan et al., 2022). Teachers’ sustained effort to have a better ERT is also indicative of their intrinsic motivation and a strong sense of responsibility that in spite of their high job burnout and dissatisfaction documented in pre-pandemic studies (e.g., Pourshahbaz, 2016), they did not walk away from their jobs and fulfilled their duties even more than what was expected from them.

Support: Despite teachers’ high sense of commitment to their job and concerns for their students’ learning, they were deprived of organizational support and strong leadership in coping with the stressors. They had just their colleagues’ assistance and guidance to acquire essential skills and knowledge to be able to continue teaching under adverse circumstances. They shared e-contents, links to useful websites, clips of their teachings, and their experience of working with helpful applications, platforms, and software. For many, this was a valuable source of gaining and developing the TPACK.

It is logical to say that teachers’ internal resources and the community’s support lowered their job burnout and inspired them to adapt to the situation instead of abandoning their attempts (Bakker et al., 2005). However, inevitably working in a non-supportive environment had a significant role in increasing job burnout (Collie et al., 2012). The teachers were frustrated because they did not feel any organizational care about their well-being, appreciation of their hard work, and support for their socio-emotional needs (Eder and Eisenberger, 2008). In contrast to some previous studies, lack of organizational support did not signify low work commitment; however, low organizational support had certainly influenced teachers’ job satisfaction (Oubibi et al., 2022).

## 7. Conclusion

The conflicting role of teachers’ ICT skills and knowledge during the pandemic as a job resource or demand has been untouched by education researchers. Inspired to fill this gap, the current study examined the interrelationship between teachers’ coping mechanisms, TPACK, and their job burnout by gathering and analyzing both quantitative and qualitative data.

The result of SEM revealed a mediating role for TPACK in the relationship between active positive coping strategies and job burnout, indicating that those teachers who used more problem-focused coping strategies had lower job burnout because they had higher TPACK. What can be induced from the results is a restorative power of TPACK on teachers’ working conditions during the pandemic on one hand, and its constructive role in assisting teachers to manage their job-related distress in that stressful condition on the other hand. From teachers’ discussion of the sources of burnout, it is concluded that technology and its associated variables, i.e., infrastructure and knowledge base, constructed the core of job burnout that in its own turn influenced students’ motivation, engagement, and learning as well as teachers’ workload and well-being. TPACK, developed by blood, sweat, and tears, was found to be the main support for teachers’ strength and resilience to hold the ERT. It is inferred that despite being deprived of organizational support, the social support from the community of teachers assisted them in achieving their work goals and professional development to some extent.

Based on the gained results, the study contributes to the literature in four ways. First, it affirms that teachers’ well-being and job security are critically interwoven with job resources and demands and strongly impacted by organizational support. Second, it reflects the fact that technology can function as a double-edged sword in teachers’ professional success and health, and incuriosity to educational technology would bring about serious consequences for educational professionals. Third, it displays that teachers’ dissatisfaction with their job conditions and the way they face and overcome their frustrations can be perceived through the strength of the link between their own and their colleagues’ technological competence and knowledge base and the availability of necessary technological infrastructures and devices. Fourth, it provides evidence of the fact that job resource is equally established in individual empowerment and organizational support and valuably co-constructed by the close and active collaboration of a professional community.

The practical implications and applications of the findings of the study for policymakers, teacher educators, and school administrators are evident. Primarily, wise and effective leadership from the MOE on formulation and execution of IT policy for technology integration into primary and secondary education is essentially required. Sequentially, the implementation of these plans should be accompanied by creating and improving technological infrastructures at schools and reinforced by suitable and continual access to technological devices for teachers, students, and parents. As for pre-service teachers, more attention should be given to TPACK courses across the syllabus of different subjects to train more technologically competent teachers. As for in-service teachers, workshops and in-service courses on useful technologies considering teachers’ needs should be offered to lessen the negative impact of technological knowledge/skill deficiency on teachers’ professional well-being and health. The value of collective wisdom in resolving the crisis in adverse conditions and events demands school administrators and principals establish teachers networks for professional support and cooperation.

## 8. Limitations and future scope

The findings of the study should be interpreted considering the limitations the researchers faced in the course of carrying out the work. First and foremost, because of the length of the questionnaires, recruiting a very large sample was impossible. Second, the data were gathered at the end of the pandemic, and the teachers were asked to complete the scales based on their whole experience of the ERT from February 2020 through a retrospective lens. Third, the context of teaching (public vs. private schools) was not a variable due to the unavailability of enough participants. Further, as the study utilized a cross-sectional design no manipulation of the variables took place. Moreover, the subjects of the study were limited to Iranian high-school teachers, and thus extrapolating the results should be done with caution. Last but not least, the study used self-report measures and due to practicality issues observational data could not be gathered.

Despite the insightful findings of this study, some research domains remain open for further investigation regarding the issue of TPACK, burnout, and coping strategies. Utilizing experimental designs or observational studies to probe into the impact of TPACK on teachers’ coping mechanisms and their job burnout is recommended. Reflecting on the results of this study, manipulating TPACK through interventions and examining the impact of TPACK instruction on teachers’ well-being and mental health as well as coping mechanisms is desired. Examining more complex relations by incorporating teachers’ individual variations (e.g., age, experience, gender, etc.) into the model is also encouraged. It would be revealing if researchers across different contexts and nations can cooperate and examine the role of TPACK in assisting teachers, of different backgrounds and cultures, to experience healthier work conditions and organizational commitment. To gain a deeper understanding of the interplay of the variables of the current study, the use of other types of qualitative data-gathering techniques such as think-aloud protocols or ethnography is suggested.

## Data availability statement

The raw data supporting the conclusions of this article will be made available by the authors, without undue reservation.

## Ethics statement

Ethical review and approval was not required for the study on human participants in accordance with the local legislation and institutional requirements. The patients/participants provided their written informed consent to participate in this study.

## Author contributions

NR carried out the study, gathered the data, and helped in writing the manuscript. MR conceptualized, designed, and supervised the research, and drafted, wrote, reviewed and edited the manuscript. All authors contributed to the article and approved the submitted version.

## Conflict of interest

The authors declare that the research was conducted in the absence of any commercial or financial relationships that could be construed as a potential conflict of interest.

## Publisher’s note

All claims expressed in this article are solely those of the authors and do not necessarily represent those of their affiliated organizations, or those of the publisher, the editors and the reviewers. Any product that may be evaluated in this article, or claim that may be made by its manufacturer, is not guaranteed or endorsed by the publisher.

## Supplementary material

The Supplementary material for this article can be found online at: https://www.frontiersin.org/articles/10.3389/fpsyg.2023.1129910/full#supplementary-material
