# Supplementary material for: Teachers’ post-pandemic outlook on the role of Technological and Pedagogical Content Knowledge in coping with burnout under adverse conditions: How a job demand transformed into a job resource
Source: Front Psychol. 2023 Mar 8;14:1129910. doi: 10.3389/fpsyg.2023.1129910 (PMC10031132; doi:10.3389/fpsyg.2023.1129910)
Supplement: Supplementary file 1 [file Data_Sheet_1.docx]

**Appendix 1- Summary of items and factor loadings from factor analysis of**

**MBI-ES**

| Items | Factor loadings | | |
| --- | --- | --- | --- |
|  | 1 | 2 | 3 |
| **Emotional Exhaustion** |  |  |  |
| I feel emotionally drained from my work as a result of the pandemic. | .703 |  |  |
| I feel used up at the end of the workday and online teaching. | .769 |  |  |
| I feel fatigued when I get up in the morning and have to face another day on the job of online teaching. | .795 |  |  |
| Working with students all day in virtual classes is really a strain for me. | .801 |  |  |
| I feel burned out from my work amid the pandemic. | .795 |  |  |
| I feel frustrated by my job due to online teaching. | .793 |  |  |
| I feel I am working too hard on my job to hold the online classes. | .646 |  |  |
| Working with people virtually puts too much stress on me. | .611 |  |  |
| I feel like I’m at the end of my rope since I teach online. | .684 |  |  |
| **Personal Accomplishment** |  |  |  |
| I can easily understand how my students feel about things via online classes. |  | .660 |  |
| I deal very effectively with the problems of my students in online classes. |  | .735 |  |
| I feel I’m positively influencing my students’ lives through my online teaching |  | .769 |  |
| I feel very energetic during online classes. |  | .714 |  |
| I can easily create a relaxed atmosphere with my students in online classes. |  | .689 |  |
| I feel exhilarated after working online with my students. |  | .601 |  |
| I have accomplished many worthwhile things in this job during the pandemic. |  | .690 |  |
| While teaching online, I deal with emotional problems very calmly. |  | .605 |  |
| **Depersonalization** |  |  |  |
| I feel I treat some students as if they were impersonal objects in online classes. |  |  | .660 |
| I’ve become more callous towards people since I teach online. |  |  | .723 |
| I worry that this way of teaching is hardening me emotionally. |  |  | .622 |
| I don’t really care what happens to some students during the pandemic. |  |  | .701 |
| I feel that students blame me for some of their problems in online classes. |  |  | .622 |
| **Variance** | **31.80** | **15.18** | **7.78** |

**Appendix 2- Summary of items and factor loadings from factor analysis of**

**TPACK-deep**

| Items | Factor loadings | | | | |
| --- | --- | --- | --- | --- | --- |
|  | 1 | 2 | 3 | 4 | 5 |
| **Design** |  |  |  |  |  |
| I can update an instructional material (paper based, electronic or multimedia materials, and etc.) based on the needs (students, environment, duration, and etc.) by using technology. | .697 |  |  |  |  |
| I can use technology to determine students’ needs to a content area in the pre-teaching process. | .697 |  |  |  |  |
| I can use technology to develop activities based on students ‘needs to enrich the teaching the teaching and learning process. | .711 |  |  |  |  |
| I can plan the teaching and learning process according to available technological resources. | .550 |  |  |  |  |
| I can conduct a needs analysis for Technologies to be used in the teaching and learning process to increase the quality of teaching. | .534 |  |  |  |  |
| I can optimize the duration of the lesson by using technologies (educational software,  virtual labs, and etc.). | .564 |  |  |  |  |
| I can develop appropriate assessment tools by using technology. | .366 |  |  |  |  |
| I can combine appropriate methods, techniques and technologies by evaluating their attributes in order to present the content effectively. | .460 |  |  |  |  |
| I can use technology to appropriately design materials to the needs for and effective teaching and learning process. | .475 |  |  |  |  |
| I can organize the educational environment in an appropriate way to use technology. | .417 |  |  |  |  |
| **Exertion** |  |  |  |  |  |
| I can implement effective classroom management in the teaching and learning process in which technology is used. |  | .425 |  |  |  |
| I can assess whether students have the appropriate content knowledge by using technology. |  | .642 |  |  |  |
| I can apply instructional approaches and methods appropriate to individual differences with the help of technology. |  | .582 |  |  |  |
| I can use technology for implementing educational activities such as homework, projects, and etc. |  | .537 |  |  |  |
| I can use technology-based communication tools (blogs, forums, chat, email, etc.) in the teaching process. |  | .650 |  |  |  |
| I can use technology for evaluating students’ achievement in related content areas. |  | .646 |  |  |  |
| I can be an appropriate model for the students in following codes of ethics for the use of technology in my teaching. |  | .606 |  |  |  |
| I can guide students in the process of designing technology-based products (presentations, games, films, and etc. |  | .570 |  |  |  |
| I can use innovative technologies (Instagram, blogs, podcasting, You Tube, and etc.) to support the teaching and learning process. |  | .558 |  |  |  |
| **Knowledge empowerment** |  |  | .717 |  |  |
| I can use technology to update my knowledge and skills in teaching English. |  |  | .761 |  |  |
| I can update my technological knowledge for the teaching process. |  |  | .753 |  |  |
| I can use technology to keep my content knowledge updated. |  |  |  |  |  |
| **Ethics** |  |  |  |  |  |
| I can provide each student equal access to technology. |  |  |  | .357 |  |
| I can behave ethically in acquiring and using special/private information –which will be used in teaching English-via technology (audio records, video records, documents, and etc.). |  |  |  | .561 |  |
| I can use technology in every phase of the teaching and learning process by considering the copyright issues (e.g., license). |  |  |  | .602 |  |
| I can follow the teaching profession’s codes of ethics in online educational environments (SHAD, Adobe Connect, Skyroom, Moodle, etc.). |  |  |  | .708 |  |
| I can provide guidance to students by leading them to valid and reliable digital sources. |  |  |  | .694 |  |
| I can behave ethically regarding the appropriate use of technology in educational environments. |  |  |  | .704 |  |
| **Proficiency** |  |  |  |  |  |
| I can troubleshoot problems that could be encountered with online educational environments (SHAD, Adobe Connect, Skyroom, Moodle, etc.). |  |  |  |  | .754 |
| I can troubleshoot any kind of problem that may occur while using technology in any phase of the teaching and learning process. |  |  |  |  | .821 |
| I can use technology to find solutions to problems (structuring, updating and relating the content to real life, etc.). |  |  |  |  | .554 |
| I can become a leader in spreading the use of technological innovations in my teaching community. |  |  |  |  | .628 |
| I can cooperate with other disciplines regarding the use of technology to solve problems encountered in the process of presenting content. |  |  |  |  | .629 |
| **Variance** | **44.95** | **5.97** | **4.18** | **3.87** | **3.17** |

**Appendix 3- Summary of items and factor loadings from factor analysis of**

**Brief-COPE**

| Items | Factor loadings | | | | | | | |
| --- | --- | --- | --- | --- | --- | --- | --- | --- |
|  | 1 | 2 | 3 | 4 | 5 | 6 | 7 | 8 |
| **Avoidant** |  |  |  |  |  |  |  |  |
| I’ve been saying to myself “this isn’t real”. | .477 |  |  |  |  |  |  |  |
| I’ve been refusing to believe that it has happened. | .417 |  |  |  |  |  |  |  |
| I’ve been using alcohol or other drugs to myself feel better. | .814 |  |  |  |  |  |  |  |
| I’ve been using alcohol or other drugs to help me get through it | .858 |  |  |  |  |  |  |  |
| I’ve been giving up trying to deal with it. | .613 |  |  |  |  |  |  |  |
| I’ve been giving up the attempt to cope. | .616 |  |  |  |  |  |  |  |
| **Active positive** |  |  |  |  |  |  |  |  |
| I’ve been concentrating my efforts on doing something about the situation I’m in. |  | .400 |  |  |  |  |  |  |
| I’ve been taking action to try to make the situation better. |  | .710 |  |  |  |  |  |  |
| I’ve been trying to see it in a different light, to make it seem more positive. |  | .551 |  |  |  |  |  |  |
| I’ve been looking for something good in what is happening. |  | .487 |  |  |  |  |  |  |
| I’ve been trying to come up with a strategy about what to do. |  | .808 |  |  |  |  |  |  |
| I’ve been thinking hard about what steps to take. |  | .333 |  |  |  |  |  |  |
| **Support** |  |  |  |  |  |  |  |  |
| I’ve been getting emotional support from others. |  |  | .686 |  |  |  |  |  |
| I’ve been getting comfort and understanding from someone. |  |  | .632 |  |  |  |  |  |
| I’ve been getting emotional support from others. |  |  | .681 |  |  |  |  |  |
| I’ve been getting comfort and understanding from someone. |  |  | .691 |  |  |  |  |  |
| **Acceptance** |  |  |  |  |  |  |  |  |
| I’ve been accepting the reality of the fact that it has happened. |  |  |  | .673 |  |  |  |  |
| I’ve been learning to live with it. |  |  |  | .676 |  |  |  |  |
| **Religion** |  |  |  |  |  |  |  |  |
| I’ve been trying to find comfort in my religion or spiritual beliefs. |  |  |  |  | .593 |  |  |  |
| I’ve been praying or meditating. |  |  |  |  | .642 |  |  |  |
| **Humor** |  |  |  |  |  |  |  |  |
| I’ve been making jokes about it. |  |  |  |  |  | .775 |  |  |
| I’ve been making fun of the situation. |  |  |  |  |  | .853 |  |  |
| **Self-blame** |  |  |  |  |  |  |  |  |
| I’ve been criticizing myself. |  |  |  |  |  |  | .706 |  |
| I’ve been blaming myself for things that happened. |  |  |  |  |  |  | .779 |  |
| **Evasive coping** |  |  |  |  |  |  |  |  |
| I’ve been turning to work or other activities to take my mind off things |  |  |  |  |  |  |  | .570 |
| I’ve been doing something to think about it less, such as going to movies, watching TV, reading, |  |  |  |  |  |  |  | .447 |
| I’ve been saying things to let my unpleasant feeling escape. |  |  |  |  |  |  |  | .479 |
| I’ve been expressing my negative feelings. |  |  |  |  |  |  |  | .330 |
| **Variance** | **23.70** | **12.67** | **5.27** | **4.89** | **4.20** | **4.02** | **3.72** | **3.69** |
